# Supplementary material for: Altered corollary discharge signaling in the auditory cortex of a mouse model of schizophrenia predisposition
Source: Nat Commun. 2023 Nov 15;14:7388. doi: 10.1038/s41467-023-42964-2 (PMC10651874; doi:10.1038/s41467-023-42964-2)
Supplement: Supplementary file 1 — Supplementary Information [file 41467_2023_42964_MOESM1_ESM.pdf]

# Supplementary Figure 1

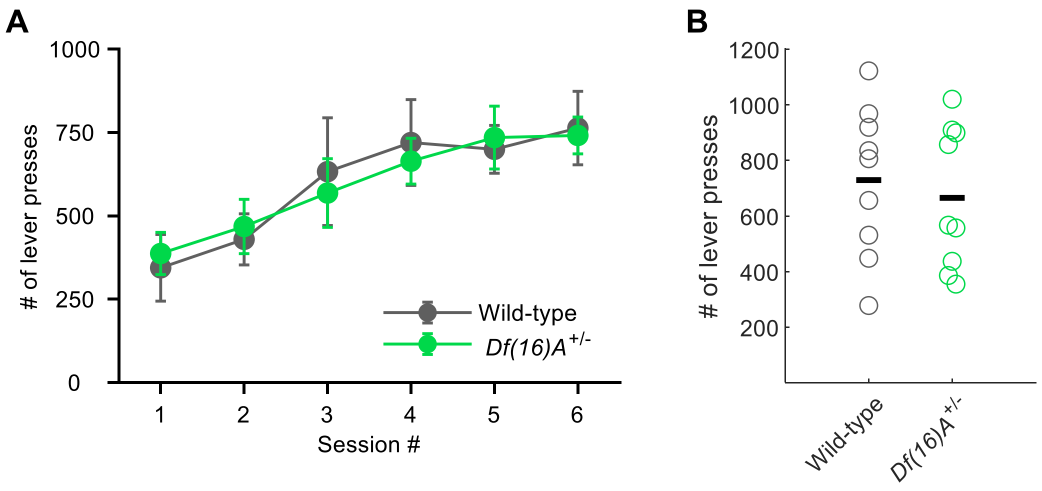


**Supplementary Figure 1. Behavioral performance during lever pressing task. A,** number of lever presses on each day of training. Error bars represent mean ± s.e.m. across animals. Performance was comparable in *Df(16)A*$^{+/-}$ and wild-type mice, as revealed by a genotype X session ANOVA (main effect of session, p = 0.001; no effect of genotype, p = 0.943; no session x genotype interaction, p =0.985). **B**, the number of self-generated sounds during the recording session was similar between genotypes (*Df(16)A*$^{+/-}$: 665.22 ± 85.34, n=9; wild-type: 729.22 ± 90.66, n=9, p=0.67, Wilcoxon two-sided rank-sum test). Horizontal black lines indicate the average across mice in each genotype. Source data are provided as a Source Data file.

# Supplementary Figure 2

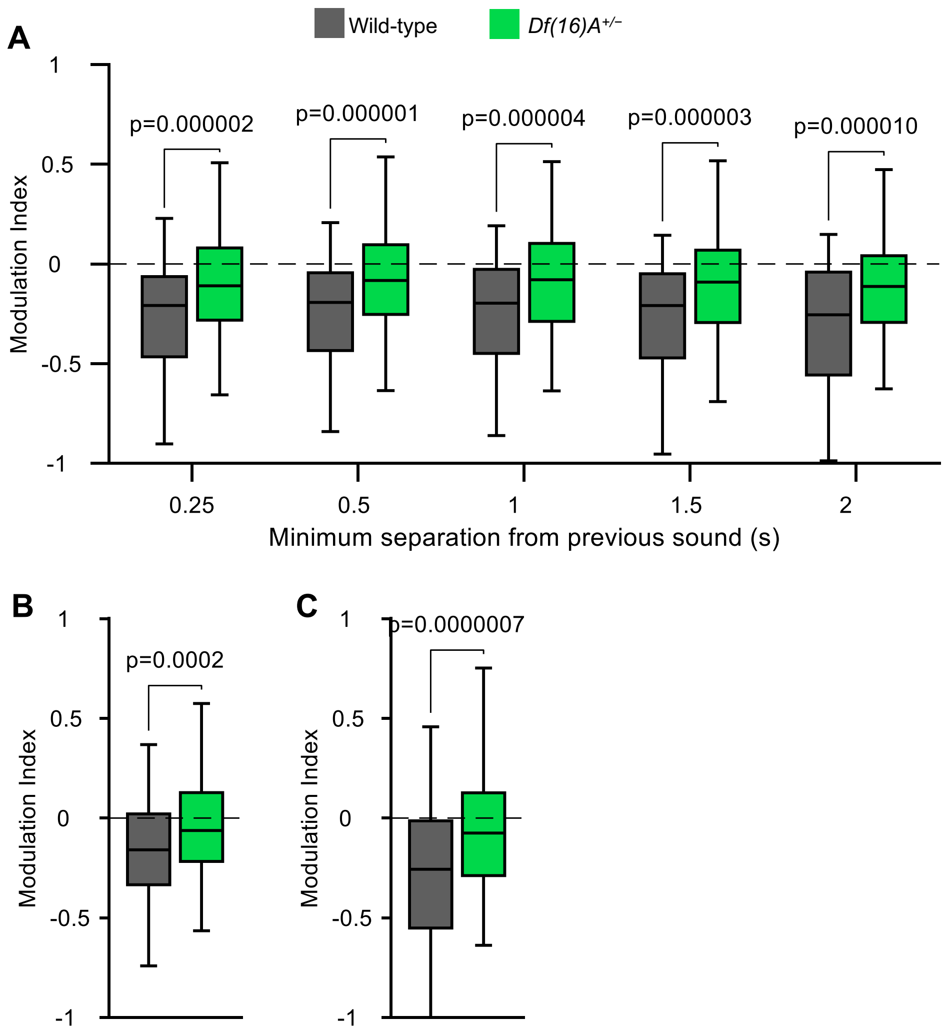


**Supplementary Figure 2. Comparison of modulation indices in *Df(16)A*$^{+/-}$ and wild-type mice using different sound selection criteria. A,** Modulation index of auditory cortex neurons recorded from *Df(16)A*$^{+/-}$ and wild-type mice after selecting random and self-generated sounds for analysis that have different minimum temporal separations from the previous sound (*Df(16)A*$^{+/-}$: n=210, 211, 197, 187 and 169 neurons; wild-type: n=203, 201, 187, 175 and 166 neurons). A minimum separation of 1s is what is used for most of the analyses in the manuscript; these results are the same as those shown in Figure 1G. (0.25: **B-C**, modulation indices calculated using random sounds occurring before (1.05 to 0.05s) a lever press (**B**; *Df(16)A*$^{+/-}$: n=185 neurons; wild-type: n=153 neurons) or while the lever was being held down (**C**; *Df(16)A*$^{+/-}$: n=192 neurons; wild-type: n=188 neurons). Minimum separation from previous sound was 1s for the analyses shown in **B-C**. Box plots represent the median (line), 25th and 75th percentiles (box), and 5th and 95th percentiles (whiskers) of the distribution of neurons in each genotype. P values were calculated using a two-sided Wilcoxon rank-sum test. Data shown is from 9 *Df(16)A*$^{+/-}$ and 9 wild-type mice. Source data are provided as a Source Data file.

# Supplementary Figure 3

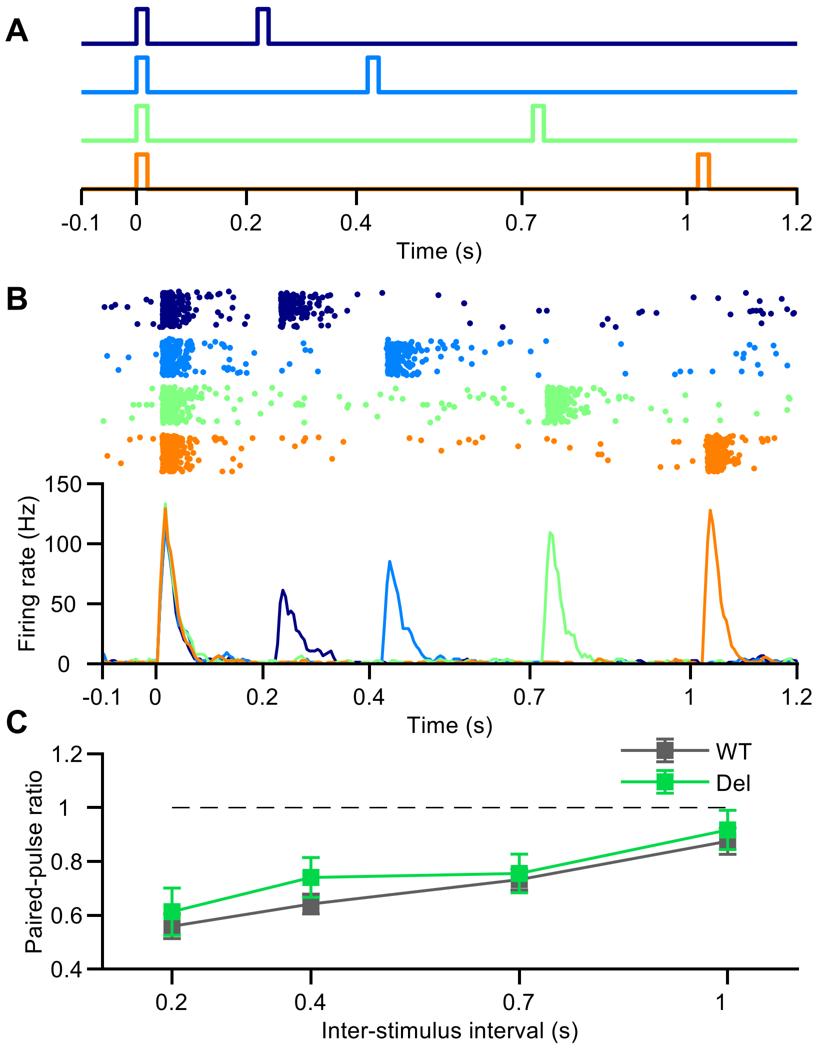


**Supplementary Figure 3. Sensory adaptation in *Df(16)A*$^{+/-}$ mice. A,** pairs of auditory stimuli were delivered at inter-stimulus intervals (ISIs) of 0.2, 0.4, 0.7 and 1 s. **B,** rasterplots (top) and PSTHs (bottom) showing the responses of an auditory cortical neuron to paired auditory stimuli at the different ISIs. **C**, The paired-pulse ratio (PPR; see Methods) at different ISIs in *Df(16)A*$^{+/-}$ mice (68 neurons from 6 mice) and wild-type mice (80 neurons from 5 mice). PPR values became larger with increasing ISIs but did not differ between genotypes, indicating that sensory adaptation is not affected in *Df(16)A*$^{+/-}$ mice (Genotype X IPI ANOVA; Main effect of IPI, $p<0.0001$, no main effect of genotype, $p=0.5328$, no interaction, $p=0.8048$). Error bars represent mean ± s.e.m. across neurons. Source data are provided as a Source Data file.

# Supplementary Figure 4

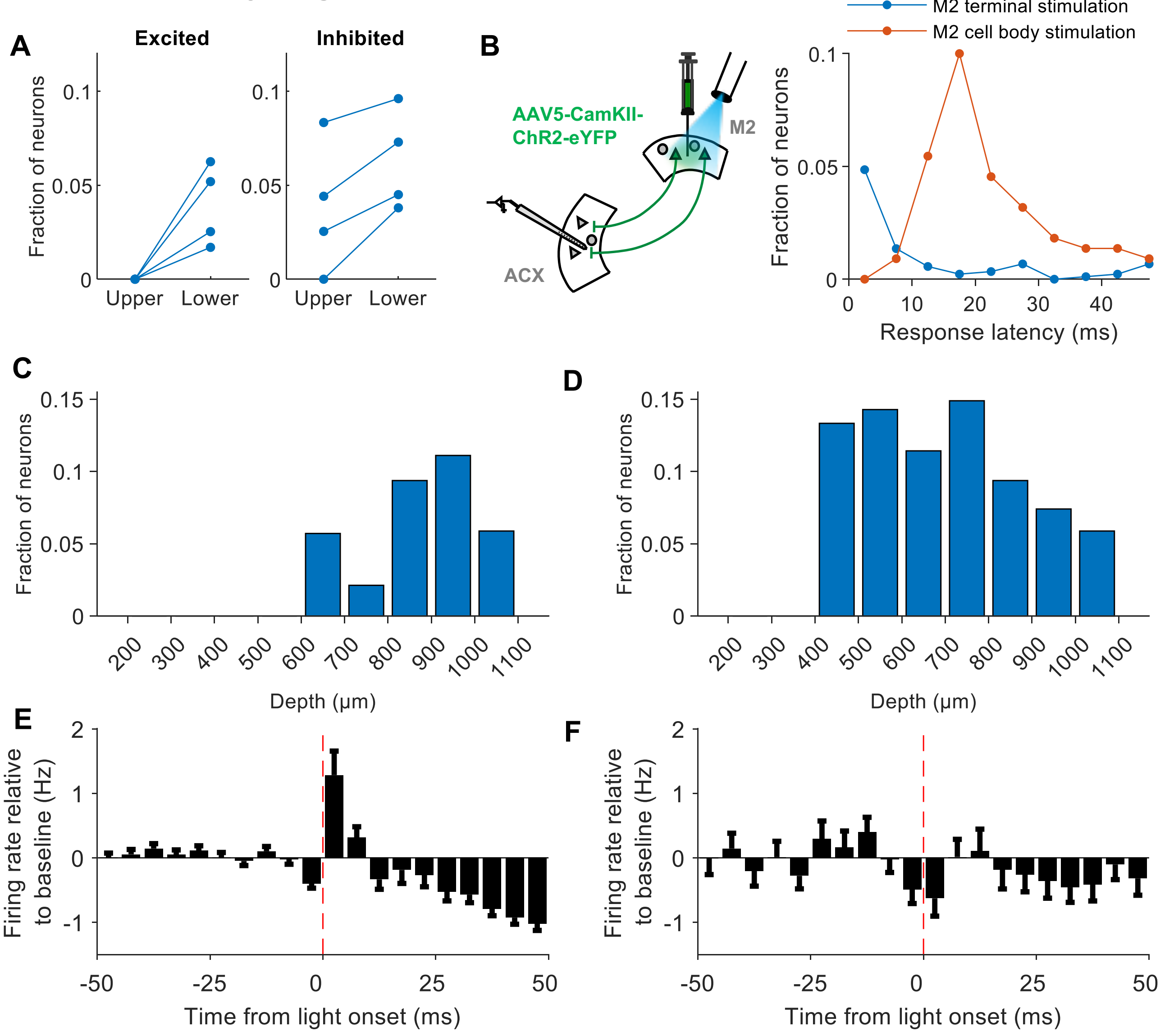


**Supplementary Figure 4. Top-down projections from the motor cortex influence activity in the auditory cortex. A,** Fraction of auditory cortex neurons in upper and lower layers in each animal showing short-latency excitatory responses (left) and inhibitory responses (right) to M2 axon terminal stimulation. **B,** a virus expressing Channelrhodopsin-2 (ChR2) was injected into M2 and the cell bodies of M2 neurons were optogenetically stimulated while at the same time recording the activity of auditory cortical neurons. The graph on the right shows the latency of excitatory responses in ACx neurons to light stimulation of M2 cell bodies (n=220 neurons) or terminals in ACx (n=886 neurons). Note the longer latency following M2 cell body stimulation, reflecting axonal conduction delays. **C,D**, fraction of auditory cortical neurons (n=220) showing short-latency excitatory responses **(C)** and inhibitory responses **(D)** to M2 cell body stimulation, as a function of their depth below the cortical surface (compare with Figure 4I and J). Fractions in **C** and **D** were calculated relative to the total number of neurons at each depth. **E-F,** Firing rates (baseline-subtracted) of all auditory cortical neurons in response to light delivered to the auditory cortex in mice with (**E**, n=886 neurons) and without (**F**, n=161 neurons) ChR2 expression in M2. Error bars represent the mean ± s.e.m. across neurons. Data shown in **A-E** is from 4 mice, in **F** from 3 mice. Source data are provided as a Source Data file.

# Supplementary Figure 5

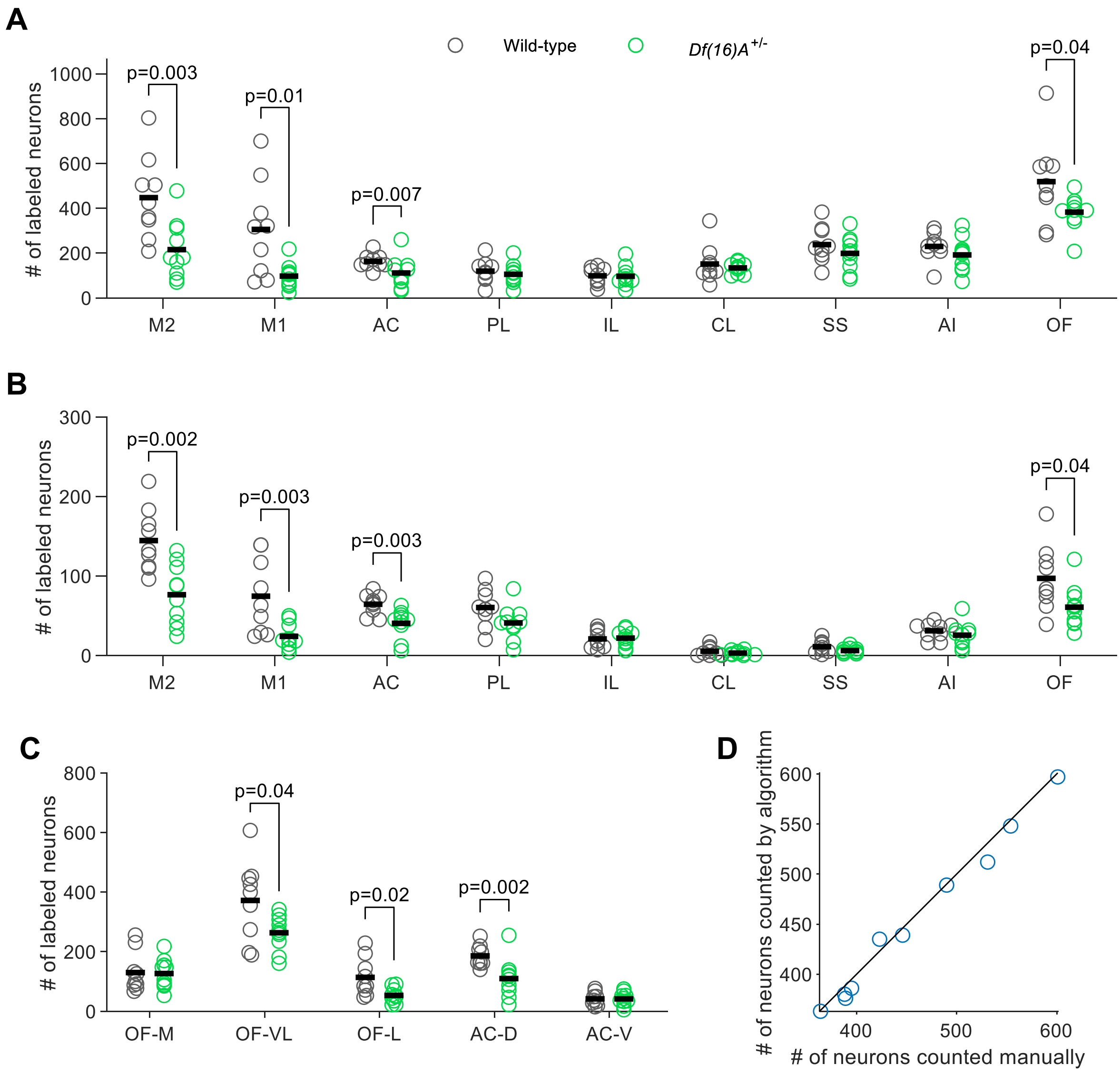


**Supplementary Figure 5. Decreased motor cortical inputs to the auditory cortex in *Df(16)A*$^{+/-}$ mice**. **A-B**, number of retrogradely labeled neurons in each frontal region in *Df(16)A*$^{+/-}$ and wild-type mice ipsilateral **(A)** and contralateral **(B)** to the injection site. **C**, Number of retrogradely labeled neurons in subregions of the anterior cingulate and orbitofrontal cortices. Horizontal black lines indicate the average number of neurons across mice in each genotype. **D**, The numbers of cells detected using our semi-automated analysis pipeline were similar to numbers obtained by manual counting. Each circle represents the cells counted in one brain section. M1, primary motor cortex; AC, anterior cingulate cortex; PL, prelimbic cortex; IL, infralimbic cortex; CL, claustrum; SS, somatosensory cortex; AI, anterior insula; OF, orbitofrontal cortex; A1, primary auditory cortex; DA, dorsal auditory cortex; VA, ventral auditory cortex; OF-M, medial orbitofrontal cortex; OF-VL, ventrolateral orbitofrontal cortex; OF-L, lateral orbitofrontal cortex; AC-D, dorsal anterior cingulate cortex; AC-V, ventral anterior cingulate cortex. P values were calculated using a two-sided Wilcoxon rank-sum test. Data in **A-C** is from 10 *Df(16)A*$^{+/-}$ and 9 wild-type mice, in **D** from 2 mice. Source data are provided as a Source Data file.
