# Supplementary material for: Altered corollary discharge signaling in the auditory cortex of a mouse model of schizophrenia predisposition
Source: Nat Commun. 2023 Nov 15;14:7388. doi: 10.1038/s41467-023-42964-2 (PMC10651874; doi:10.1038/s41467-023-42964-2)
Supplement: Supplementary file 3 — Reporting Summary [file 41467_2023_42964_MOESM3_ESM.pdf]

nature portfolio

Corresponding author(s): Torfi Sigurdsson

Last updated by author(s): Oct 4, 2023

Reporting Summary

Nature Portfolio wishes to improve the reproducibility of the work that we publish. This form provides structure for consistency and transparency in reporting. For further information on Nature Portfolio policies, see our Editorial Policies and the Editorial Policy Checklist.

Statistics

For all statistical analyses, confirm that the following items are present in the figure legend, table legend, main text, or Methods section.

n/a	Confirmed	
☐	☒	The exact sample size (*n*) for each experimental group/condition, given as a discrete number and unit of measurement
☐	☒	A statement on whether measurements were taken from distinct samples or whether the same sample was measured repeatedly
☐	☒	The statistical test(s) used AND whether they are one- or two-sided<br>*Only common tests should be described solely by name; describe more complex techniques in the Methods section.*
☒	☐	A description of all covariates tested
☐	☒	A description of any assumptions or corrections, such as tests of normality and adjustment for multiple comparisons
☐	☒	A full description of the statistical parameters including central tendency (e.g. means) or other basic estimates (e.g. regression coefficient) AND variation (e.g. standard deviation) or associated estimates of uncertainty (e.g. confidence intervals)
☐	☒	For null hypothesis testing, the test statistic (e.g. *F*, *t*, *r*) with confidence intervals, effect sizes, degrees of freedom and *P* value noted<br>*Give P values as exact values whenever suitable.*
☒	☐	For Bayesian analysis, information on the choice of priors and Markov chain Monte Carlo settings
☒	☐	For hierarchical and complex designs, identification of the appropriate level for tests and full reporting of outcomes
☒	☐	Estimates of effect sizes (e.g. Cohen's *d*, Pearson's *r*), indicating how they were calculated

Our web collection on statistics for biologists contains articles on many of the points above.

Software and code

Policy information about availability of computer code

Data collection	Electrophysiological data were acquired using RHD2000 Interface software, Intan Technologies
Data analysis	Clustering of action potential waveforms was performed using Klusta (https://github.com/kwikteam/klusta)<br>Semi-automatic counting of neurons was performed using ImageJ<br>Alignment of neurons to brain areas was performed using WholeBrain (www.wholebrainsoftware.com)<br>Further analysis was performed using custom-written scripts in Matlab.

For manuscripts utilizing custom algorithms or software that are central to the research but not yet described in published literature, software must be made available to editors and reviewers. We strongly encourage code deposition in a community repository (e.g. GitHub). See the Nature Portfolio guidelines for submitting code & software for further information.

Data

Policy information about availability of data

All manuscripts must include a data availability statement. This statement should provide the following information, where applicable:

- Accession codes, unique identifiers, or web links for publicly available datasets
- A description of any restrictions on data availability
- For clinical datasets or third party data, please ensure that the statement adheres to our policy

Source data are provided with this paper. Raw data are available from the corresponding author upon reasonable request.

Human research participants

Policy information about studies involving human research participants and Sex and Gender in Research.

Reporting on sex and gender	N/A
Population characteristics	N/A
Recruitment	N/A
Ethics oversight	N/A

Note that full information on the approval of the study protocol must also be provided in the manuscript.

Field-specific reporting

Please select the one below that is the best fit for your research. If you are not sure, read the appropriate sections before making your selection.

- [x] Life sciences
- [ ] Behavioural & social sciences
- [ ] Ecological, evolutionary & environmental sciences

For a reference copy of the document with all sections, see nature.com/documents/nr-reporting-summary-flat.pdf

Life sciences study design

All studies must disclose on these points even when the disclosure is negative.

Sample size	No statistical methods were used to predetermine sample size, but our sample sizes (number of animals) are similar to previous studies (comparison of neuronal activity during behavior between genotypes, e.g. PMID 20360742, 28869582 and 33077947; comparison of anatomical connectivity between genotypes, e.g. PMID 25913858, 31079872; optogenetic characterization of projections, e.g. PMID 24005287). Sample sizes were chosen to ensure robustness of findings while at the same time keeping the number of experimental animals to the necessary minimum, in accordance with 3R principles.
Data exclusions	Animals injected with virus that failed to show expression were excluded.
Replication	Due to the time-intensive nature of the experiments, data was collected sequentially from several cohorts of animals, each of which contained approximately the same number of animals in each experimental group. All analysis was performed on the combined data across cohorts. Due to the relatively low sample sizes in each cohort, we did not attempt to replicate findings across cohorts.
Randomization	The experimental groups in the study consist of knockout mice and their wild-type litter-mate controls and could therefore not be randomly assigned. However, care was taken that each experimental cohort contained approximately the same numbers of knockout and wild-type animals.
Blinding	For all experiments involving genotype comparisons investigators were blinded to genotype during data collection. Investigators were furthermore blinded to genotype during analyses that were partly performed manually (spike sorting and semi-automatic counting of retrogradely labeled neurons across brain areas). All subsequent analysis steps were fully automated and therefore did not require blinding of investigators.

Reporting for specific materials, systems and methods

We require information from authors about some types of materials, experimental systems and methods used in many studies. Here, indicate whether each material, system or method listed is relevant to your study. If you are not sure if a list item applies to your research, read the appropriate section before selecting a response.

Materials & experimental systems

n/a	Involved in the study	
[ ]	[x]	Antibodies
[x]	[ ]	Eukaryotic cell lines
[x]	[ ]	Palaeontology and archaeology
[ ]	[x]	Animals and other organisms
[x]	[ ]	Clinical data
[x]	[ ]	Dual use research of concern

Methods

n/a	Involved in the study	
[x]	[ ]	ChIP-seq
[x]	[ ]	Flow cytometry
[x]	[ ]	MRI-based neuroimaging

Antibodies

Antibodies used	Rabbit anti-GFP antibody (Invitrogen, Catalog #A11122)<br>Secondary anti-rabbit fluorescent antibody (488 nm; Invitrogen, Catalog #A11008)
Validation	Validation is provided by datasheets on the manufacturer's website, which also lists relevant publications that have previously used the antibodies:<br>Rabbit anti-GFP antibody: https://www.thermofisher.com/antibody/product/GFP-Antibody-Polyclonal/A-11122<br>Secondary anti-rabbit fluorescent antibody: https://www.thermofisher.com/antibody/product/Goat-anti-Rabbit-IgG-H-L-Cross-Adsorbed-Secondary-Antibody-Polyclonal/A-11008

Animals and other research organisms

Policy information about studies involving animals; ARRIVE guidelines recommended for reporting animal research, and Sex and Gender in Research

Laboratory animals	Df(16)A+/− mice<br>C57BL/6N mice<br>All mice were 7-16 weeks old at the beginning of the experiments and were housed in individual cages inside a ventilated animal cabinet (Scantainer, Scanbur). Ambient temperature and humidity were maintained at 20-24 C and 40-65%, respectively. Animals were maintained on a 12-h light/dark cycle (lights on at 8 a.m.) and all experiments were performed during the light phase.
Wild animals	No wild animals were used in the study
Reporting on sex	Due to the time-intensive nature of the experiments and limited resources, only male animals were used in the study and a comparison between sexes was not performed.
Field-collected samples	No field-collected samples were used in the study
Ethics oversight	All procedures were approvide by the local animal care committee (License: TVA FU-1256; Regierungspräsidium Darmstadt, Germany)

Note that full information on the approval of the study protocol must also be provided in the manuscript.
